# Halotolerant Endophytic Fungi: Diversity, Host Plants, and Mechanisms in Plant Salt–Alkali Stress Alleviation

**DOI:** 10.3390/plants14182907

**Published:** 2025-09-18

**Authors:** Qiurui Ma, Yangyuxin Liu, Zi Liu, Yang Xu, Shuren Yin, Helong Bai, Jing Wang

**Affiliations:** College of Chemistry, Changchun Normal University, Changchun 130032, China; 13578927109@163.com (Q.M.); lyyx24@outlook.com (Y.L.); 18943539435@163.com (Z.L.); 2409010212@stu.ccsfu.edu.com (Y.X.); 15560604768@163.com (S.Y.)

**Keywords:** halotolerant endophytic fungi, salt–alkali stress tolerance, plant–microbe interactions, biostimulants, ecological adaptation

## Abstract

Halotolerant endophytic fungi (HEFs) represent a critical biological resource in mitigating plant salt–alkali stress, demonstrating remarkable adaptability across diverse ecological environments. This comprehensive review analyzes 150 scientific publications, revealing HEFs’ multifaceted mechanisms of plant stress tolerance. Inhabiting over 30 host plant species without causing pathogenic effects, these fungi enhance plant resilience through sophisticated physiological strategies. Key findings highlight HEFs’ ability to modulate ionic homeostasis, elevate antioxidant capacities, and stimulate plant growth under saline conditions. The research unveils the potential of HEF metabolites as biostimulants and explores their co-evolutionary hypotheses with host plants. Despite promising laboratory and field validations, significant challenges remain in HEFs’ practical agricultural applications, including environmental factor interactions and biotechnological ethical considerations. Future research directions emphasize deeper investigations into HEFs’ ecological adaptability and microbiological interactions to unlock their full agricultural potential.

## 1. Introduction

In the global landscape of land resources, salinization has emerged as a critical challenge that demands urgent attention. Soil is defined as being saline when the electrical conductivity (EC) of the saturation extract (ECe) in the root zone exceeds 4 dSm^−1^ at 25 °C and has an exchangeable sodium of 15% (*w*/*v*). Salinization also includes excessive accumulation of ions such as calcium (Ca^2+^), magnesium (Mg^2+^), sodium (Na^+^), sulphates (SO_4_^2−^), and chlorides (Cl^−^) in the soil, inhibiting plant growth and cellular functions. The most abundant ion in most salt-affected soils is Na+, and hence the exchange phase is dominated by Na^+^ [1]. A December 2024 report by the Food and Agriculture Organization (FAO) revealed that over 1.381 billion hectares of land worldwide—accounting for 10.7% of the planet’s total area—are suffering from salinization. Particularly severe impacts are observed in regions including Australia, Argentina, Kazakhstan, and China. As climate change intensifies, the accelerating pace of land salinization is delivering devastating blows to agricultural production, with crop yields in affected areas declining by an average of over 30% [2].

The accumulation of salt in the soil caused by salinization will reduce yields, tree survival rates, and affect land use strategies [3]. High salt levels can lead to ionic toxicity, osmotic stress, oxidative damage, and other secondary stresses in plants [4]; therefore, plants need to maintain the balance of ions in their cells to ensure normal growth and development. The large areas of saline–alkali land in inland China mainly contain three cations: Na^+^, K^+^, Mg^2+^, and four anions: CO_3_^2−^, HCO_3_^−^, Cl^−^, and SO_4_^2−^ [5]. Excessive accumulation of Na^+^ and Cl^−^ can lead to ionic toxicity, which reduces the absorption of essential nutrients such as calcium (Ca), potassium (K), phosphorus (P), and nitrogen (N) [6]. Further deterioration will lead to widespread plant dieback, furthering soil desertification and seriously threatening the security of the environmental ecosystem [7]. Although the plant itself initiates a series of physiological and molecular stress responses to mitigate salinity damage, these defense mechanisms are insufficient to cope with increasing salinity stress.

In this context, using plant growth-promoting fungi to enhance crop productivity has emerged as a novel strategy for addressing salinity stress. Endophytic fungi, a specialized group of microbial enhancers, have garnered significant attention in recent years. Fungal endophytes are symbiotic microorganisms that are often found in asymptomatic plants [8]. The halophilic endophytic fungi discussed in this article are a group of microorganisms that can survive stably in saline–alkali environments (soil electrical conductivity ECe > 4 dS/m, pH 7.5–9.5, tolerating 50–300 mM NaCl or pH 8.0–9.0 alkaline conditions). These non-pathogenic fungi colonize various host plants through metabolic symbiosis, forming specific mechanisms such as producing osmotic regulators, regulating ion homeostasis, enhancing antioxidant systems, and modulating plant hormones. These mechanisms collectively alleviate salt stress and improve the host’s stress resistance. These microorganisms reside within plants, functioning as their “second genome”—serving as vital agents that help host plants thrive while resisting both biological and abiotic stresses [8,9]. Endophytic fungi have established a unique symbiotic relationship with host plants. They not only do not cause obvious pathogenic damage to host plants, but also actively help plants adapt to harsh abiotic environments, such as salt and alkali [4].

Endophytic fungi cultivate symbiotic relationships through the production of secondary bioactive metabolites. Within these ecosystems, these microorganisms not only obtain essential nutrients and protective mechanisms from host plants but also release bioactive stimulants to promote plant growth and development. In prolonged saline–alkaline environments, plants and endophytic fungi develop a synergistic resistance mechanism through genetic co-selection and metabolic complementarity. This dynamic partnership effectively regulates plant physiological functions while enhancing their stress tolerance capabilities [5,6].

**Figure 1:**
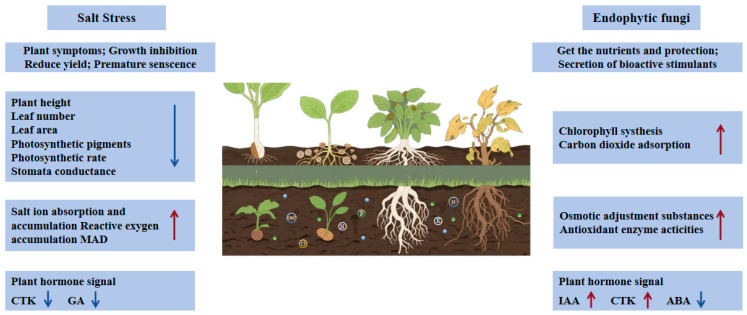
Effects of salt stress on plants and the enhancement of salt tolerance by endophytic fungi.

Numerous studies have demonstrated the existence of complex networks of mutualistic symbiosis among microorganisms. As shown in Figure 1, endophytic fungi enhance plant nutrient uptake efficiency and precisely regulate developmental processes and physiological functions by secreting signaling compounds, plant hormones, or hormone analogs, thereby activating the host plant’s stress resistance potential [10,11,12,13,14]. Endophytic fungi have a very close relationship with their hosts, and can even biosynthesize the same chemicals as their hosts, such as indole-3-acetic acid in *Cynodon dactylon*, geniposide in *Trifolium repens* L., and paclitaxel in *Taxus baccata Ericoides*. These substances help endophytic fungi better integrate into the microenvironment of plant tissues and jointly cope with salt and alkali stress [15,16,17]. Endophytic fungi occupy a unique ecological niche, and their relationship with host plants maintains a delicate and dynamic balance between mutualistic symbiosis, parasitism, and cohabitation, which is crucial for the survival and reproduction of plants in a saline–alkali environment.

To comprehensively investigate the role and mechanisms of salt-tolerant endophytic fungi in enhancing plant salt–alkali tolerance, we conducted a systematic literature review using keywords including “salt-alkali tolerance”, “response to salt-alkali stress”, “salt tolerance mechanisms”, “alkaline-tolerant microorganisms”, and “fungi”. The search was performed through databases such as Wiley and Spring, with 150 relevant articles extensively reviewed. Among these, 55 studies specifically focused on endophytic fungi’s contributions to improving plants’ salt–alkali tolerance capabilities.

Based on the comprehensive literature review, this study will first systematically examine the host range and diversity of salt-tolerant endophytic fungi, revealing their distribution patterns and ecological adaptations across various plant hosts. Subsequently, we will conduct a detailed comparative analysis of isolation and identification techniques for these fungi, establishing a systematic framework to elucidate the fundamental mechanisms underlying the “fungus-plant synergy” in stress resistance.

## 2. Diversity and Host Specificity

Up to now, salt-tolerant endophytic fungi have been isolated and identified from more than 50 species of 42 genera and 30 families in different saline–alkali habitats around the world (coastal tidal flats, inland salt marshes, arid saline–alkali land, etc.), covering two ecological groups of wild halophytes and cultivated crops, and the host coverage is about 2.8 times higher than that of ordinary endophytic fungi [4]. Endophytic salt-tolerant fungi exhibit an extensive host spectrum, having been isolated from various plants distributed across diverse saline–alkali soil habitats worldwide, including both cultivated crops and wild halophytes, as shown in Table 1. This section systematically summarizes their host plant range to elucidate the adaptive mechanisms of these fungi towards different plant species and their ecological distribution patterns.

Salt-tolerant wild plants, primarily inhabiting extreme saline–alkali environments such as coastal mudflats, inland salt marshes, and arid saline–alkali lands, include species like Suaeda and Stargrass. These plants have evolved distinctive morphological adaptations and physiological mechanisms to withstand environmental stresses, thereby providing stable ecological niches for salt-tolerant endophytic fungi.

In cultivated crops, salt-tolerant endophytic fungi have been found colonizing the roots of various major food and cash crops, including *Triticum aestivum* L., *Oryza sativa*, *Gossypium hirsutum*, *Solanum lycopersicum,* etc., which are widely distributed in agricultural areas around the world. The soil salinity of their colonization environment is usually lower than that of wild plant habitats, but they can still be isolated and obtained in local salinized areas.

It follows that a complex ecological interaction exists between salt-tolerant endophytic fungi and their host plants [4]. On one hand, the species of host plants, their ecological habits, and the salinity characteristics of their habitats significantly influence the community composition and distribution patterns of fungi, demonstrating the habitat filtering effect. On the other hand, salt-tolerant endophytic fungi that colonize these hosts enhance the growth performance and salt tolerance of the host plants through their physiological metabolic activities, indicating potential for co-evolution between them [18,19]. Future studies should further analyze the interaction specificity between different host plants and specific salt-tolerant endophytic fungi, as well as the dynamic law of these interactions in relation to the ecological environment [18].

**Table 1:** Common endogenous fungi and host plants.

Crops	Floristics	Geographical Distribution	Soil pH	Electrical Conductivity	Sodium Adsorption Ratio	The Main Fungal Genera Isolated	References
Wilding	*Robinia pseudoacacia* L.	100° E, 35° N	6.3	5.1	8.9	*Fusarium*	[20]
Wilding	*Elaeagnus angustifolia* Linn.	124° E, 43° N	8.4	9.3	15.2	*Fusarium*	[21]
Wilding	*Puccinellia tenuiflora*	110° E, 38° N	9.0	11.2	18.7	*Fusarium*	[18,22]
Wilding	*Arundo donax* L.	70° E, 40° N	8.3	5.4	7.9	*Fusarium*	[20]
Wilding	*Setaria viridis*	124° E, 43° N	8.2	9.4	14.3	*Fusarium*	[20]
Wilding	*Saussurea japonica (Thunb.) DC.*	102° E, 35° N	7.5	3.2	8.8	*Penicillium*	[23]
Wilding	*Anthemis nobilis*	90° E, 55° N	7.2	4.1	8.1	*Penicillium*	[24]
Wilding	*Suaeda salsa*	126° E, 35° N	9.2	10.3	13.7	*Penicillium*	[25]
Wilding	*Arabidopsis thaliana* (L.) *Heynh.*	Europe, western Asia	6.8–7.5	3.9	7.3	*Penicillium*	[26]
Wilding	*Spartina anglica Hubb*	100° E, 44° N	6.5–8.5	6.8	7.2	*Fusarium*	[26]
Wilding	*Lolium perenne* L.	106° E, 26° N	6.5	4.1	6.5	*Aspergillus*	[27]
Wilding	*Festuca elata Keng ex E. B. Alexeev*	102° E, 40° N	8.5	6.2	8.5	*Neotyphodium*	[26]
Wilding	*Populus* L.	90° E, 45° N	6.5–8.0	6.7	5.1	*Penicillium*	[25]
Wilding	*Trifolium repens* L.	Europe and West Asia	6.0–7.0	4.5	12.5	*Alternaria arborescens*	[16]
Wilding	*Glycyrrhiza uralensis Fisch.*	123° E, 44° N	7.8	8.2	5.6	*Fusarium*	[28]
Wilding	*Medicago sativa* L.	105° E, 45° N	6.5–7.5	5.0	6.3	*Fusarium*	[25]
Cultivated	*Solanum lycopersicum*	125° E, 43° N	6.0–7.0	5.1	7.1	*Fusarium oxysporum*	[20]
Cultivated	*Zea mays* L.	125° E, 43° N	6.0–7.5	3.9	5.8	*Fusarium oxysporum*	[21]
Cultivated	*Cucumis*	125° E, 43° N	5.5–7.0	7.2	7.6	*Fusarium oxysporum*	[26]
Cultivated	*Triticum aestivum* L.	Subtropical	6.0–7.5	4.9	9.8	*Fusarium oxysporum*	[29]
Cultivated	*Gossypium hirsutum*	102° E, 47° N	6.0–8.0	8.1	5.3	*Fusarium oxysporum*	[30]
Cultivated	*Arachis hypogaea*	120° E, 40° N	5.6–6.0	7.5	11.2	*Fusarium*	[26]
Cultivated	*Oryza sativa*	122° E, 45° N	8.2	5.2	13.8	*Fusarium*	[27]
Cultivated	*Beta vulgaris* L.	122° E, 45° N	6.0–6.7	5.9	7.9	*Fusarium oxysporum*	[30]
Cultivated	*Cucumis melo* L.	122° E, 45° N	6.0–7.5	5.4	6.8	*Fusarium oxysporum*	[30]

## 3. Isolation and Identification Techniques

The identification and isolation of endophytic fungi resistant to salinity is the basis for studying their diversity and functions. The combination of traditional methods and high-throughput technology provides a multi-dimensional tool for analyzing fungal–plant interactions.

Traditional cultivation methods rely on artificial media for the growth, isolation, and purification of endophytic fungi, but often fail to capture their diverse populations due to incomplete cultivation. The rapid advancement of genetic sequencing technologies, particularly high-throughput sequencing (HTS), has revolutionized this field. Modern molecular biology approaches now provide groundbreaking tools for studying endophytic fungal diversity.

### 3.1. Separation Method

**Figure 2:**
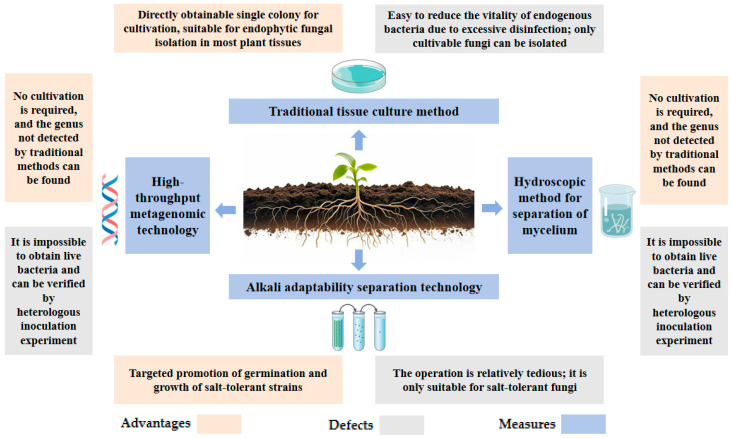
Comparison of advantages and disadvantages of separation methods.

The halophilic endophytic fungi require the sterile treatment of host tissues and the preservation of fungal vitality. The core is to eliminate surface microbial contamination and promote the growth of endogenous bacteria. The advantages and disadvantages of the four separation methods are shown in Figure 2.

#### 3.1.1. Traditional Tissue Culture Method

Host plant tissues (e.g., roots, stems, leaves) are first rinsed with sterile water, then soaked in 75% ethanol for 30–60 s. Subsequently, they are treated with a 1% sodium hypochlorite solution for 5–10 min, followed by 3–5 rinses with sterile water to remove residual disinfectants. The processed tissues are cut into 0.5–1 cm segments and inoculated onto PDA (potato glucose agar) or MEA (malt extract agar) media containing antibiotics (e.g., penicillin, streptomycin). The cultures are incubated under dark conditions at 25–28 °C for 3–7 days. Once fungal hyphae emerge from the tissue edges, the tip hyphae are isolated and transferred to fresh medium for purification, yielding single colonies [23,30].

However, this method may lead to reduced viability of endogenous bacteria due to excessive disinfection. For example, in the isolation of the halophilic endophytic fungi from halophytes, the treatment time of sodium hypochlorite should be optimized to adapt to the effect of high salinity on tissue permeability [21].

#### 3.1.2. Alkali Adaptability Separation Technology

For fungi isolated from halophyte roots, adding gradient concentrations of NaCl (e.g., 50–200 mM) or adjusting pH to 8.0–9.0 in culture media can simulate the saline–alkaline environment of host growth, thereby promoting the germination and development of salt-tolerant strains. For instance, when isolating the phosphorus-depleting fungus Apophysomyces spartima, adding 1.15% NaCl to the culture medium significantly enhanced its survival rate [21,31].

#### 3.1.3. Hydroscopic Method for Separation of Mycelium

First, add the sample (such as soil or rhizosphere material) to an appropriate amount of sterile water and stir to disperse it. The mixture is then filtered through sieves with different mesh sizes in sequence: smaller impurities pass through while larger components like mycelium are retained. Subsequently, rinse the sieves with sterile water to remove residual contaminants. Finally, collect the mycelium for further purification and identification procedures [32].

#### 3.1.4. High-Throughput Metagenomic Technology

By directly extracting total DNA from plant tissues, high-throughput sequencing (e.g., Illumina MiSeq, Illumina, San Diego, CA, USA) is performed using fungal-specific fragments (e.g., ITS region), combined with bioinformatics analysis (e.g., OTU clustering and species annotation) to decipher the composition of endophytic fungal communities. For instance, in wheat root endophytes research, metagenomic technology has identified rare fungal genera undetected by traditional culture methods (e.g., Pseudogymnoascus), though their functions require validation through heterologous inoculation experiments [21].

### 3.2. Identification Techniques: From Morphological Characteristics to Molecular Phylogenetic Analysis

#### 3.2.1. Morphological Identification

To perform preliminary classification, observe colony morphology (color, texture, marginal characteristics), hyphal structure (presence of septa and branching patterns), and spore morphology (shape, size, and attachment method) by referring to the “Fungal Identification Manual”. For example, *Penicillium* colonies typically appear bluish-green with broom-like, branched conidiophores, while *Aspergillus* species possess terminal sacs and radiating conidiophore heads [21]. However, this method has limited ability to distinguish morphologically similar species, and some fungi do not produce spores under culture conditions, making them difficult to identify.

#### 3.2.2. Molecular Identification

Using fungal genomic DNA as a template, ITS sequences were amplified through PCR (primers ITS1: TCCGTAGGTGAACCTGCGG and ITS4: TCCTCCGCTTATTGATATGC), with taxonomic identification conducted via GenBank database alignment and phylogenetic tree analysis [21]. For closely related species, supplementary identification using genetic markers such as β-tubulin and tef1α is required. For instance, distinguishing *Penicillium* from *Aspergillus* requires phylogenetic analysis of the β-tubulin gene in addition to ITS for enhanced resolution [21]. Sampling timing and experimental cycles must also be carefully considered: endophytic fungi may significantly promote host development during early growth stages, while others play crucial roles in enhancing host stress resistance during later phases. Ma Wei’s team employed 454 sequencing technology for metagenomic analysis of endophytic fungi in ginseng seeds, revealing their diversity and interspecies distribution patterns [33]. The Mariani team classified four polyphyletic groups of *Fusarium* endophytes isolated from wild Indonesian red bananas based on morphological characteristics and ITS sequences. Soltani’s team identified 11 fungal taxa in Mediterranean juniper (*Cupressus sempervirens* L.) through PCR amplification and nuclear ITS rDNA sequencing. Researchers further performed systematic taxonomic identification of these 11 fungal taxa using NCBI nucleotide database retrieval tools.

## 4. Functional Validation: From Lab to Field

The functional validation of salt-tolerant endophytic fungi requires a systematic evaluation from controlled environment experiments (lab/greenhouse) to field applications. Laboratory-level functional validation is fundamental for revealing the role of HEFs, with its core lying in accurately assessing their enhancement of plant salt–alkali tolerance through controlled variable methods. Current mainstream experimental designs need to consider salt stress type (neutral salt/alkaline salt), concentration gradients, and the systematic measurement of host plant physiological response indicators. This section integrates 55 key studies (2018–2023), revealing the actual effects and translational bottlenecks of fungal–plant interactions by comparing physiological indicator responses under different experimental designs.

### 4.1. Experimental Designs

Pot and hydroponic experiments precisely control salt gradients (typically 50–300 mM NaCl), pH (7.5–9.0), and fungal inoculation concentrations (10^5^−10^7^ spores/g substrate) to quantify the improvement effects of endophytic fungi on plant physiology, the effect of improvement. Table 2 summarizes changes in key indicators for 15 typical fungal–plant combinations under salt stress. For example, *Penicillium simplicissimum* (10^6^ spores/g) reduced Na^+^ accumulation in wheat roots by 38%, increased the K^+^/Na^+^ ratio by 2.1 times, and simultaneously activated the proline synthetase gene P5CS expression, resulting in a 67% increase in leaf proline content [34]. Similarly, rice inoculated with *Aspergillus terreus* showed a 45% increase in photosynthetic rate under 200 mM NaCl, directly related to fungal-secreted cyclic peptide metabolites activating the SOS1 sodium efflux pump [35]. Notably, fungal colonization rates in laboratory environments generally reach 70–90%, but high salt (>250 mM) can reduce colonization efficiency by 30–50% [36,37].

**Figure 3:**
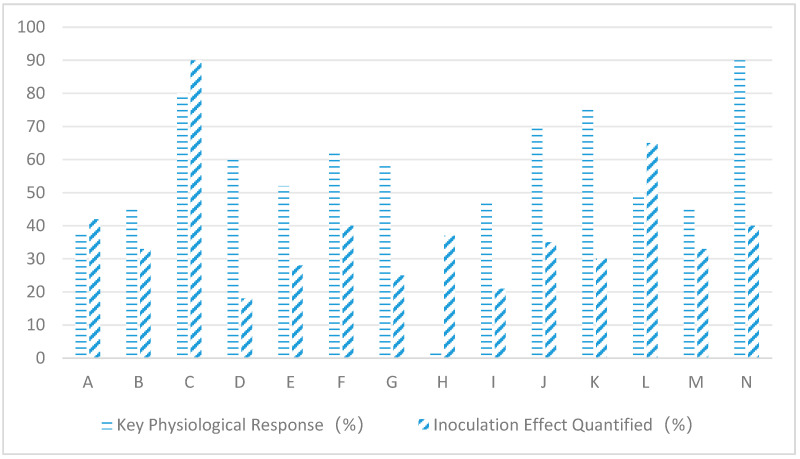
Effects of different salinity gradients on main indicators of host plants. Note: A: Wheat + Penicillium simplicissimum; B: Rice + Aspergillus terreus; C: Seepweed + Alternaria alternata; D: Cotton + Fusarium oxysporum; E: Maize + Trichoderma harzianum; F: Tomato + Epicoccum nigrum; G: Soybean + Chaetomium globosum; H: Alfalfa + Phoma glomerata; I: Sunflower + Cladosporium cladosporioides; J: Goji Berry + Talaromyces wortmannii; K: Sugar Beet + Fusarium redolens; L: Cucumber + Trichoderma asperellum; M: Sorghum + Curvularia lunata; N: Alkali Grass + Sarocladium strictum.

As can be seen from Figure 3, endogenous fungal inoculation has a significant effect on the main physiological indexes of host plants under different salt stress conditions. When wheat was paired with *Penicillium simplicissimum*, sodium ion levels decreased by 38% and biomass increased by 42%; rice combined with *Aspergillus* terreus boosted photosynthetic rate by 45% and tillering number by 33%; Suaeda japonica integrated with *Alternaria alternata* enhanced superoxide dismutase by 80% and plant survival rate by 90%; cotton coexisted with *Fusarium oxysporum*, resulting in 55% increased IAA in root systems and 18% elongation of fiber length; corn paired with *Trichoderma harzianum* reduced malondialdehyde by 50% and increased grain yield by 28%; tomato combined with *Epicoccum nigrum* decreased hydrogen peroxide by 62% and fruit set rate by 40%; soybean integrated with *Chaetomium glibosum* elevated proline content by 58% and protein concentration by 25%; alfalfa paired with *Phoma glomerata* enhanced aquaporin gene expression by 2.4% and dry matter accumulation by 37%; sunflower combined with *Cladosporium cladoaporiodes* improved stomatal conductance by 48% and seed oil content by 21%; and barbary wolfberry integrated with *Talaromyces wortmannii* enhanced ion partitioning efficiency by 70% and carotenoid production by 35%. When sweet potatoes are combined with *Fusarium redolens*, soluble sugar levels increase by 75% and tuber sugar content rises by 30%. Cucumbers paired with *Trichoderma asperellum* show 50% higher lignin precipitation and 65% reduced wilt incidence. Sorghum treated with *Curvularia lunata* demonstrates 45% decreased sodium ion adsorption while achieving 33% improved water use efficiency. Algae mixed with *Sarocladium strictum* boosts organic acid secretion by 90% and increases salt gland density by 40%.

### 4.2. Field Trials

**Figure 4:**
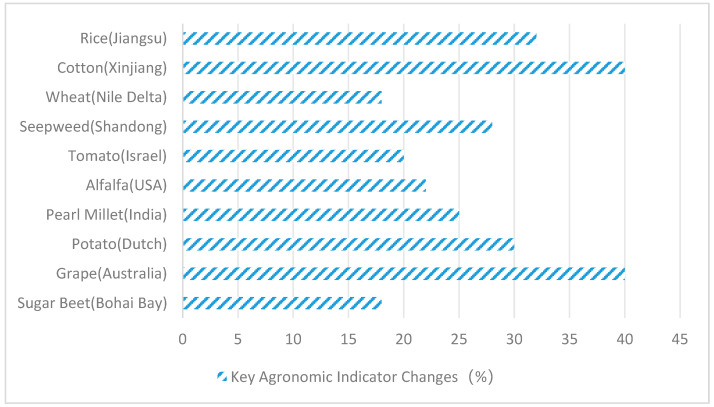
Changes in key agronomic indicators (%).

Field trials focus on practical application effects in saline–alkali lands (EC_e_ > 4 dS/m, pH 8.5–9.5). Table 3 compares 10 representative studies, showing that fungal inoculation can increase crop yield by 15–40%, but the effect is modulated by environmental factors. For example, *Aspergillus flavus* increased rice yield by 32% in coastal saline soil (pH 8.7), but its colonization rate decreased from 85% in the lab to 52% in the field [35]. Composite inoculants (e.g., *Penicillium* + *Trichoderma*) exhibit greater stability: in Xinjiang saline–alkali cotton fields (pH 9.2), dual inoculation increased cotton fiber yield by 40% and maintained rhizosphere microbial diversity by secreting cyclic hexapeptides (cyclo-(L-Pro-L-Tyr)) [50,51]. Figure 4 clearly shows the change in key indicators. Current bottlenecks lie in the attenuation of colonization efficiency in the field (typically 30–50%) and disruption of metabolite synthesis by environmental fluctuations (e.g., temperature fluctuations causing a 60% decrease in flavonoid derivative production), which need to be addressed through synthetic biology modification of fungi or the development of encapsulated delivery systems [52].

As can be seen from the comparative analysis in Figure 4, fungi and plants form a synergistic symbiosis. Although the yield increase is inconsistent under different environmental factors, both have significant effects and strong stability. As Figure 4 demonstrates, in Jiangsu Province, rice cultivation with *Aspergillus flavus* increased yields by 32%; in Xinjiang, cotton paired with *P. simplicissimum* + *T. harzianum* boosted fiber production by 40%; in the Nile River region, wheat combined with *Fusarium verticillioides* raised thousand-grain weight by 18%; in Shandong, *Suaeda japonica* integrated with *Alternaria alternata* enhanced biodiesel output by 28%; in Israel, tomatoes paired with *Epicoccum nigrum* increased soluble solids by 20%; in the United States, alfalfa combined with *Phoma glomerata* elevated crude protein by 22%; in India, pearl millet paired with *Curvularia lunata* boosted grain yield by 25%; in Henan, potatoes with *Sarocladium strictum* improved tuber starch content by 30%; in Australia, grapes with *Talaromyces wortmannii* increased resveratrol levels by 40%; and in the Bohai Bay region, sugar beets with *Fusarium redolens* raised sugar content by 18%.

Furthermore, beyond the well-documented synergy of Penicillium + Trichoderma, other composite inoculants have demonstrated significant synergistic benefits in high pH–saline soils. For instance, the combination of *Aspergillus terreus* and *Fusarium oxysporum* enhanced rice yield by approximately 30% under pH 9.0 conditions by concurrently improving ion homeostasis and antioxidant capacity. Similarly, *Alternaria alternata* and Cladosporium cladosporioides co-inoculation markedly increased the survival rate and biomass of Suaeda salsa in the Yellow River Delta by promoting organic acid secretion and Na^+^ compartmentalization. Additionally, the pairing of *Epicoccum nigrum* and *Sarocladium strictum* has proven effective in solanaceous crops under high pH stress (pH 9.0–9.5), primarily through phytohormone modulation and enhanced water-use efficiency. These examples underscore the potential of tailored fungal consortia in mitigating saline–alkali stress across diverse agroecological contexts.

## 5. Mechanisms of Fungal-Mediated Alleviation of Saline–Alkali Stress

### 5.1. Physiological Adaptations: Osmolyte Production, Ion Exclusion, Antioxidant Defense, and Phytohormone Modulation

Saline–alkali stress imposes dual constraints on plants: (1) osmotic stress via disrupted cellular water potential gradients, and (2) ionic toxicity from excessive cytosolic Na^+^ accumulation coupled with K^+^ deficiency.

Halotolerant endophytic fungi (HEFs)—defined as fungi that colonize plant tissues asymptomatically and thrive in saline–alkaline environments—mitigate these constraints through specialized mechanisms distinct from general endophytes, mycorrhizal fungi, or lichen symbionts.

#### 5.1.1. Osmolyte Production: Fungal vs. Host-Derived Compounds

**Figure 5:**
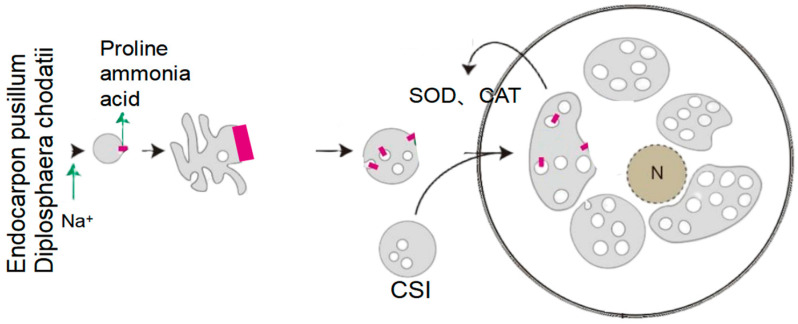
Key mechanism of induced accumulation of complementary solutes.

HEFs employ a bipartite strategy for osmotic adjustment:

Fungal-derived osmolytes: HEFs directly synthesize compatible solutes to stabilize cellular turgor (see Figure 5):

Aspergillus montevidensis *ZYD4* accumulates glycerol and trehalose under salt stress, with transcriptomics revealing upregulation of *GPD1* (glycerol-3-phosphate dehydrogenase) and *TPS1* (trehalose-6-phosphate synthase) [32].

Trichoderma afroharzianum *T22* secretes mannitol (a polyol), which is translocated to host roots, enhancing water retention under 300 mM NaCl [32].

Synergistically, the mycobiont *E. pusillum* secretes *D-mannitol* (C_6_H_14_O_6_), which is translocated to *D. chodatii* via hyphal networks. This polyol enhances cellular viability to 78.3% under extreme saline–alkali conditions, suggesting a division of labor in osmotic protection [32].

Saprophytic fungi similarly drive osmolyte production. *Clitopilus hobsonii* upregulates host Δ^1^-pyrroline-5-carboxylate synthase (P5CS; EC 2.7.2.11) expression 2.3-fold, increasing soluble sugar content by 41% (65% sucrose/glucose) in *Liquidambar styraciflua* under K^+^ limitation (0.05 mM) [53]. Notably, fungal *ChACU* (a K^+^ transporter) expression correlates positively with host proline accumulation (R^2^ = 0.89), implicating K^+^ signaling in osmotic metabolism regulation [53].

Mycorrhizal fungi employ “dual regulation”: *Rhizophagus irregularis* induces host proline accumulation (1.6-fold increase) while synergizing with biochar to enhance soil water-holding capacity by 28%. This combination maintains leaf relative water content at 82% (vs. 59% in controls) and preserves chlorophyll (SPAD index = 39.5) [54]. Mechanistically, fungal-secreted indole-3-acetic acid (*IAA*) triggers abscisic acid (*ABA*) synthesis via 9-cis-epoxycarotenoid dioxygenase (*NCED3*; EC 1.13.11.51) activation, establishing a “fungal signal → hormonal cascade → osmolyte accumulation” axis [54].

Host-induced osmolytes: HEF trigger host biosynthesis of proline, glycine betaine, and soluble sugars:

*T. afroharzianum T22* elevates root proline in Pisum sativum by 169% via ABA-mediated activation of Δ^1^-pyrroline-5-carboxylate synthase (P5CS; EC 2.7.2.11) [54].

Clitopilus hobsonii induces Liquidambar styraciflua to produce sucrose/glucose (65% of total soluble sugars) via P5CS upregulation, a plant-mediated response distinct from fungal osmolytes [54,55,56].

#### 5.1.2. Ion Exclusion Mechanisms: Na^+^/K^+^ Balance Regulation

HEFs maintain ion homeostasis through species-specific strategies, contrasting with mycorrhizal or saprophytic fungi:

Active Na^+^ Exclusion

*Trichoderma harzianum T22* secretes apigenin (C_15_H_10_O_5_), a fungal-derived flavonoid that directly binds *SOS1* (Na^+^/H^+^ antiporter) with high affinity (K_D_ = 1.8 μM), increasing Na^+^ efflux by 58% [54]. This bypasses the canonical *SOS3-SOS2* kinase cascade, unlike mycorrhizal *Rhizophagus irregularis*, which relies on ABA-responsive elements (*ABREs*) in the *SOS1* promoter [54].

*Piriformospora indica* enhances host *HKT1* (high-affinity K^+^ transporter) and *KAT1/KAT2* (K^+^ channels), reducing Na^+^/K^+^ ratios in *Arabidopsis thaliana* under salt stress [57].

Selective K^+^ Acquisition

*Clitopilus hobsonii* (a saprophytic fungus) expresses high-affinity K^+^ transporters (*ChACU ATPase*, ChHAK carrier) under K^+^ deficit. However, HEFs like *P. indica* exhibit strain-specific K^+^ uptake efficiency, with some isolates upregulating K^+^ channels 2–3-fold more than others [57].

**Figure 6:**
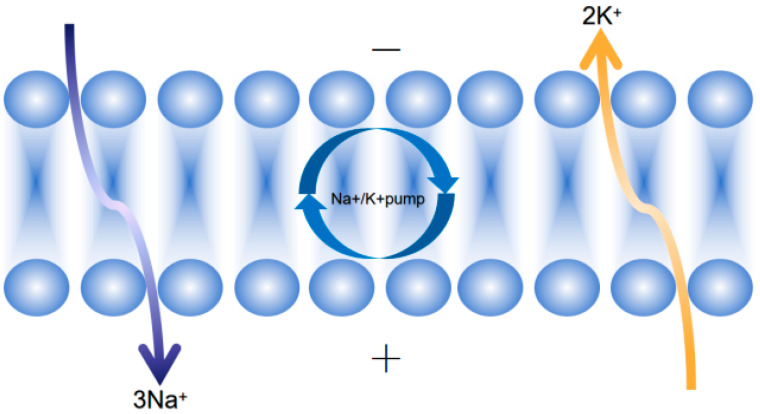
Fungi regulate Na^+^/K^+^ balance under salt and alkali stress by driving active Na^+^ exudation and K^+^ influx mechanism driven by Na^+^/K^+^-ATPase.

*Hortaea werneckii* (a black yeast HEF) uses MACPF-aegerolysin pores (0.4 nm diameter) to selectively exclude Na^+^ (hydrated radius 0.36 nm) while permitting K^+^ influx (hydrated radius 0.33 nm)—a mechanism that ensures cytosolic K^+^ retention to maintain enzymatic function under salt stress [32]. Additionally, this fungus upregulates specific superoxide dismutase (*SOD*) isoforms, including cytosolic *Cu/Zn-SOD* and mitochondrial *Mn-SOD*, to scavenge reactive oxygen species (*ROS*) generated by saline–alkali stress, further complementing its ion homeostasis strategy (see Figure 6).

#### 5.1.3. Antioxidant Enzyme Enhancement

HEFs upregulate host antioxidant enzymes to mitigate oxidative damage:

*T. afroharzianum T22* increases superoxide dismutase (*SOD*; EC 1.15.1.1) activity 1.8-fold and catalase (*CAT*; EC 1.11.1.6) activity 1.5-fold in Pisum sativum, reducing malondialdehyde (*MDA*) by 42% [58].

Aspergillus terreus enhances ascorbate peroxidase (*APX*; EC 1.11.1.11) and glutathione reductase (*GR*; EC 1.6.4.2) in rice, scavenging H_2_O_2_ and maintaining redox balance under 200 mM NaCl [59].

#### 5.1.4. Phytohormone Modulation

HEFs manipulate host phytohormone signaling to coordinate stress responses, with nuanced interactions between auxin and abscisic acid (*ABA*) pathways:

Indole-3-acetic acid *(IAA)*: While *IAA* and *ABA* often exhibit antagonistic crosstalk in plant development, *HEFs* such as *T. afroharzianum* secrete *IAA* that modulates *ABA* signaling in a stress-specific context. Specifically, fungal *IAA* may subtly enhance the expression of host *NCED3* (9-cis-epoxycarotenoid dioxygenase; EC 1.13.11.51)—a key enzyme in *ABA* biosynthesis—though this effect is secondary to the primary regulation of *NCED3* by osmotic stress itself [50].

*ABA*: Fungal-induced *ABA* (primarily triggered by osmotic stress) activates P5CS (a rate-limiting enzyme in osmolyte biosynthesis) and *SOS1* (a plasma membrane Na^+^/H^+^ antiporter), thereby linking phytohormone signaling to downstream osmotic adjustment and ion homeostasis [43].

Strigolactones: *Fusarium verticillioides* colonizing wheat induces host strigolactone secretion, which recruits rhizobia to enhance N_2_ fixation. This illustrates hormone-mediated multitrophic interactions that complement primary stress-alleviating pathways [43].

### 5.2. Molecular Mechanisms: Signaling Networks and Metabolic Reprogramming

Fungal-mediated tolerance involves integrated signaling cascades, with *SOS* pathways, *MAPK* phosphorylation, and metabolite-mediated cross-kingdom communication as core nodes.

#### 5.2.1. SOS Pathway Regulation

The conserved “Ca^2+^ → *SOS3* → *SOS2* → *SOS1*” cascade is targeted by fungi:

*Rhizophagus irregularis* secretes an aspartate-rich effector (*RiPEP1*) that binds *SOS3*’s EF-hand domain (K_D_ = 2.3 μM), stabilizing the *SOS3-SOS2* complex (half-life extended 2.1-fold). Rhizosphere acidification (ΔpH = −0.8) enhances the proton gradient for *SOS1*-mediated efflux [54].

*Trichoderma harzianum T22* induces 4-coumarate-CoA ligase (*4CL*; EC 6.2.1.12) 3.2-fold, increasing apigenin/luteolin levels. Apigenin relieves *SOS1* autoinhibition (K_D_ = 1.8 μM), boosting Na^+^/H^+^ exchange 2.3-fold [57].

*Endocarpon pusillum* activates *D. chodatii*’s mechanosensitive Ca^2+^ channel (*DcMCA*), elevating cytosolic Ca^2+^ 3-fold. This activates calmodulin (*DcCaM*), which recruits fungal *EpSOS2* to phosphorylate host *SOS1*, increasing Na^+^ efflux 1.9-fold [32].

#### 5.2.2. MAPK-Transcription Factor Networks

MAPK cascades coordinate stress responses via transcription factor activation:

In *C. hobsonii*-*L. styraciflua* symbiosis, low K^+^ upregulates *MAPK3/6* (1.7–2.0-fold). Phosphorylated *bZIP62* binds *AKT1’s ABRE* promoter element, increasing K^+^ channel expression 2.5-fold while suppressing vacuolar Na^+^ transporter *NHX7* (38% reduction) [53].

*Trichoderma afroharzianum T22* induces *PsMAPK4* (2.3-fold), activating *WRKY70*. This transcription factor upregulates *SOS1* (2.1-fold) and promotes strigolactone secretion, recruiting *Rhizobium leguminosarum* and enhancing N_2_ fixation (*nifA/nifH* upregulated 1.7–1.9-fold) [57] (see Table 4).

### 5.3. Halotolerant Endophytic Fungi: Definition and Mechanistic Distinctions

Revised Definition: Halotolerant endophytic fungi (HEFs) are endophytes that colonize plant tissues asymptomatically and enhance host salt–alkali tolerance through:

Osmolyte production: HEFs (e.g., *A. montevidensis ZYD4*) synthesize glycerol/trehalose, whereas general endophytes rely on host-derived osmolytes.

Ion exclusion mechanisms: HEFs (e.g., *P. indica*) use fungal-secreted effectors (apigenin, peptides) to activate *SOS1*, unlike non-halotolerant endophytes.

Antioxidant enzyme enhancement: HEFs (e.g., *T. afroharzianum*) upregulate SOD/CAT/APX, a trait absent in many general endophytes.

Phytohormone modulation: HEFs (e.g., *T. harzianum*) secrete *IAA* and manipulate ABA/strigolactone signaling, coordinating stress responses.

## 6. Bioactive Metabolites and Their Applications

Halotolerant endophytic fungi (HEFs) enhance plant salt tolerance through a dual strategy: by secreting their own bioactive metabolites and by inducing the accumulation of protective compounds in the host plant. Clarifying the origin of these compounds is crucial for understanding the underlying mechanisms and their application potential. This section systematically summarizes key metabolites, categorizing them based on their proven or putative source, and outlines their functional mechanisms and prospective applications (Table 5).

## 7. Summary

Saline–alkali stress impairs plant growth and development. Halophilic endophytic fungi (HEFs) enhance host plants’ salt tolerance through multiple mechanisms, including ion regulation, enhanced antioxidant capacity, and secretion of bioactive metabolites, demonstrating unique value in saline–alkali soil agricultural improvement. This symbiotic relationship between fungi and plants not only promotes crop growth and yields but also reduces chemical inputs by optimizing soil microecology, aligning with sustainable development principles. However, the application of HEFs faces challenges such as host specificity, field colonization efficiency constrained by soil conditions, and unclear interaction mechanisms with other microorganisms. Moreover, significant differences in their effectiveness across different plants severely limit large-scale implementation.

This comprehensive review examines salt-tolerant endophytic fungi, highlighting the remarkable adaptability of Heterotrichium elegans (HEF) as a vital biological resource for mitigating plant stress from salinity and alkali conditions across diverse ecosystems. Through analysis of 150 scientific studies, researchers demonstrate that HEFs can parasitize over 30 host plant species without causing pathogenic effects. The fungus enhances plant stress resistance through physiological strategies, including ion homeostasis regulation, improved antioxidant capacity, and stimulation of growth under salt stress conditions.

In addition, the bioactive metabolites secreted by HEFs and their application potential are also introduced. Meanwhile, the challenges to its field application, such as soil colonization efficiency, synthetic biology ethical controversy, and knowledge gaps in interaction mechanisms with the microbiome, are identified, so as to promote the core role of HEFs in the agricultural transformation of saline–alkali land.

## 8. Prospects and Challenges

The application of salt-tolerant endophytic fungi (HEFs) in the remediation of saline–alkali soils and sustainable agricultural development holds broad prospects. However, several core challenges must be overcome to realize their full potential.

Currently, most salt-tolerant endophytic fungi isolated from moderately saline–alkali environments lack strains adapted to high pH-saline soils. This highlights the urgent need to screen and validate high-efficiency strains (HEFs) in extreme saline–alkali conditions. Moreover, due to host specificity and environmental adaptability differences among various HEFs, developing composite microbial communities tailored for diverse crops and saline–alkali gradients has become crucial. Investigating synergistic effects and adaptation mechanisms through mixed inoculation studies is equally vital. The significant variations in HEF community structures under saline–alkali conditions demand systematic research on their diversity patterns and ecological interactions with soil physicochemical properties and host species. Although existing studies predominantly focus on crop-related fields, attention to interactions between key species in saline–alkali remediation and HEFs remains insufficient. Future research should prioritize such collaborations to provide theoretical support for ecological restoration in saline–alkali regions, transforming HEFs from resource advantages into practical productivity drivers for agricultural quality improvement and ecological enhancement in saline–alkali areas.

Despite their benefits, the application of HEFs may entail certain risks and trade-offs. For instance, some fungal strains could exhibit context-dependent behaviors, such as becoming opportunistic pathogens under stress conditions or competing with beneficial native microbiota. There is also the possibility of unintended ecological impacts, including altered soil microbial community structure or reduced biodiversity following large-scale inoculation. Additionally, the over-reliance on specific HEF strains may lead to reduced genetic diversity in agricultural systems, potentially increasing vulnerability to future biotic and abiotic stresses. Therefore, comprehensive risk assessments—including long-term field monitoring, ecological impact studies, and evaluations under varying climatic conditions—are essential to ensure the safe and sustainable application of HEF technologies.

Future efforts should also address the socio-economic aspects, such as cost-effectiveness, farmer acceptability, and scalability of HEF-based interventions, to facilitate their integration into mainstream agricultural practices.

## Figures and Tables

**Table 2:** Effects of endogenous fungal inoculation on physiological responses of host plants under salt stress.

Host Plant	Endophytic Fungus	Salt Stress Conditions	Stress Damage Markers	Adaptive Responses	Inoculation Effect Quantified	References
Wheat	*Penicillium simplicissimum*	150 mM NaCl		Na^+^ ↓38%, K^+^/Na^+^ ↑2.1×, Proline ↑67%	Biomass ↑42%	[34]
Rice	*Aspergillus terreus*	200 mM NaCl		Photosynthetic rate ↑45%, SOS1 gene ↑3.2×	Tillering number ↑33%	[35]
Seepweed	*Alternaria alternata*	300 mM NaCl		Superoxide dismutase (SOD) ↑80%	Survival rate ↑90%	[38]
Cotton	*Fusarium oxysporum*	100 mM NaCl		Root IAA ↑55%, Biofilm stability ↑60%	Fiber length ↑18%	[39]
Maize	*Trichoderma harzianum*	250 mM NaCl	MDA ↓52%	Chlorophyll ↑39%	Grain yield ↑28%	[36]
Tomato	*Epicoccum nigrum*	150 mM NaCl	H_2_O_2_ ↓62%	APX activity ↑75%	Fruit setting rate ↑40%	[40]
Soybean	*Chaetomium globosum*	200 mM NaCl		Proline ↑58%, Nodule nitrogenase ↑3.5×	Protein content ↑25%	[41]
Alfalfa	*Phoma glomerata*	100 mM NaCl		Na^+^/K^+^ ↓1.8×, Aquaporin gene ↑2.4×	Dry matter accumulation ↑37%	[42]
Sunflower	*Cladosporium cladosporioides*	200 mM NaCl		Stomatal conductance ↑48%, ABA ↓43%	Seed oil content ↑21%	[43]
Goji Berry	*Talaromyces wortmannii*	300 mM NaCl		Betaine ↑120%, Ionic compartmentalization efficiency ↑70%	Carotenoids ↑35%	[44]
Sugar Beet	*Fusarium redolens*	150 mM NaCl		Sucrose synthase ↑2.1×, Soluble sugars ↑75%	Root sugar content ↑30%	[45]
Cucumber	*Trichoderma asperellum*	100 mM NaCl		Lignin deposition ↑50%, Vessel density ↑30%	Fusarium wilt incidence ↓65%	[46]
Sorghum	*Curvularia lunata*	250 mM NaCl		Rhizosphere pH ↓0.8 units, Na^+^ adsorption ↓45%	Water use efficiency ↑33%	[47]
Arabidopsis	*Acremonium strictum*	150 mM NaCl		SOS2 gene ↑4.5×, Ion efflux ↑60%	Flowering advanced by 7 days	[48]
Alkali Grass	*Sarocladium strictum*	300 mM NaCl		Organic acid secretion ↑90%, Na^+^ chelation ↑80%	Salt gland density ↑40%	[49]

Note: ↑/↓ indicate increase/decrease relative to the non-inoculated control group; “×” indicates fold change. Physiological context: This table distinguishes between indicators of stress-induced damage (e.g., MDA, H_2_O_2_) and measures of the plant’s adaptive physiological responses (e.g., osmolyte accumulation, antioxidant enzyme activity, ion homeostasis). The alleviation of salt–alkali stress by endophytic fungi is often manifested by a reduction in damage markers and a concomitant enhancement of adaptive mechanisms, as detailed in Section 5 (Mechanisms of Fungal-Mediated Alleviation of Saline–Alkali Stress).

**Table 3:** Effects of salt-tolerant endophytic fungi on agronomic traits and economic benefits of crops in field.

Location/Soil Type	Crop	Endophytic Fungus	Treatment Method	Key Agronomic Indicator Changes	Economic Benefit	References
Jiangsu Coastal Saline Soil (pH 8.7)	Rice	*Aspergillus flavus*	Seed coating	Yield ↑32%, Empty grains rate ↓40%	Net profit ↑USD 220/ha	[35]
Xinjiang Saline–Alkali Soil (pH 9.2)	Cotton	*P. simplicissimum + T. harzianum*	Root drenching	Fiber yield ↑40%, Lint percentage ↑15%	Water saving ↑25%	[50]
Nile Delta, Egypt (pH 8.9)	Wheat	*Fusarium verticillioides*	Foliar spray	1000-grain weight ↑18%, Protein ↑12%	Nitrogen fertilizer ↓30%	[51]
Yellow River Estuary Wetland, Shandong (pH 8.5)	Seepweed	*Alternaria alternata*	Rhizosphere inoculation	Biodiesel output ↑28%, Na^+^ enrichment ↑50%	Marginal cost ↓USD 15/ton	[38]
Negev Desert, Israel (pH 9.0)	Tomato	*Epicoccum nigrum*	Drip irrigation	Soluble solids ↑20%, Fruit cracking rate ↓60%	Market price ↑15%	[40]
Central Valley, CA, USA (pH 8.6)	Alfalfa	*Phoma glomerata*	Seed pelleting	Crude protein ↑22%, Overwintering survival rate ↑35%	Harvest cycle shortened by 10 days	[42]
Saline Soil, Gujarat, India (pH 9.1)	Pearl Millet	*Curvularia lunata*	Furrow application	Grain yield ↑25%, Water requirement ↓20%	Marginal return ↑USD 45/ha	[47]
Dutch Polder Area (pH 8.4)	Potato	*Sarocladium strictum*	Seed tuber soaking	Tuber starch ↑30%, Scab ↓70%	Storage loss ↓25%	[49]
Murray-Darling Basin, Australia (pH 8.8)	Grape	*Talaromyces wortmannii*	Drip + Inoculant	Sugar-acid ratio optimized ↑1.8×, Resveratrol ↑40%	Premium wine price ↑30%	[44]
Bohai Bay Coastal Saline Soil (pH 8.7)	Sugar Beet	*Fusarium redolens*	Root dipping at transplanting	Sugar content ↑18%, Brown spot ↓55%	Processing efficiency ↑20%	[45]

Note: ↑/↓ indicate increase/decrease relative to the non-inoculated control group; “×” indicates fold change.

**Table 4:** Fungal metabolites act as signaling molecules and bioeffectors.

Metabolite	Function	Example
Siderophores	Fe^3+^ chelation (logK = 32)	Harzianic acid increases leaf Fe by 36%, reducing ROS accumulation [57].
Cyclopeptides	SOS1 autoinhibition relief (K_D_ = 1.2 μM)	cyclo(L-Phe-L-Pro) enhances Na^+^ efflux 2.3-fold [60].
Organic acids	Metal chelation/compartmentalization	A. niger citrate (12 mM) sequesters Zn^2+^/Pb^2+^ [61].
Trehalose	Protein stabilization via H-bonding	Maintains RuBisCO activity at 85% of control under K^+^ limitation [53].

**Table 5:** Bioactive metabolites involved in fungal-mediated salt tolerance and their applications.

Compound Category/Structure	Source/Origin	Functional Mechanism	Potential Patent Application Direction	References
**Fungal-Derived Metabolites**				
Siderophores (e.g., Ferrichrome)	Fungal secretion	Chelate Fe^3+^ (logK = 32) to alleviate iron deficiency; inhibit pathogen growth.	Biocontrol agent (CA2887654)	[62]
Exopolysaccharides (EPS)	Fungal secretion	Form rhizosphere biofilm, reducing Na^+^ permeation; improve soil aggregate stability.	Microbial encapsulation material (DE102019117890)	[62]
Cyclopeptides (e.g., cyclo(L-Phe-L-Pro))	Fungal secretion	Act as signaling molecules; relieve SOS1 autoinhibition (K_D_ = 1.2 μM), enhancing Na^+^ efflux.	Bioeffector compound	[58]
Fungal terpenoids (e.g., Trichoderins)	Fungal secretion	Induce plant stress response genes (e.g., OsNHX1); enhance vacuolar Na^+^ compartmentalization.	Genetic engineering promoter element (WO202209876)	[63]
Chitinase	Fungal secretion	Degrade pathogen cell walls; induce plant systemic resistance (ISR).	Biopesticide (US10709123)	[62]
**Fungal-Induced Plant Metabolites**				
Proline	Fungal-induced plant synthesis	Osmotic adjustment, maintaining cell water potential; protects protein structure and function.	Biostimulant composition (CN107384123A)	[64,65]
Betaine (Glycine betaine)	Fungal-induced plant synthesis	Osmoprotection, stabilizes photosynthetic complexes; enhances Rubisco activity.	Foliar spray for saline–alkali soil crops (US20200154621)	[52,66]
Flavonoid derivatives (e.g., Quercetin)	Fungal-induced plant synthesis	ROS scavenging, membrane stabilization; upregulate plant SOD, CAT gene expression.	Crop stress resistance enhancer (WO202112345)	[58,59]
Polyamines (Putrescine, Spermidine)	Fungal-induced plant synthesis	Inhibit ethylene synthesis; maintain DNA stability, delay senescence.	Seed coating agent (AU2018300567)	[67,68]
**Metabolites of Dual or Uncertain Origin**				
γ-Polyglutamic acid (γ-PGA)	Mainly from associated *Bacillus* spp.	Promotes rhizosphere probiotics colonization; reduces leaf Na^+^.	Soil amendment (JP2020156789)	[69]
Silicic acid polymer	Fungal-enhanced plant uptake/assimilation	Enhances cell wall silicification, reduces Na^+^ influx; increases plant SOD activity.	Silicon fertilizer technology (RU2017145678)	[43,47]
Melatonin	Putative: Fungal-induced or co-synthesis	Protects chloroplast ultrastructure; upregulates photosynthetic genes PsbA, PsbD.	Stress mitigation formulation (EP3456218)	[52,66]

## Data Availability

The data that support the findings of this study are available from the corresponding author upon reasonable request.

## Abbreviations

The following abbreviations are used in this manuscript:

HEFs	Halotolerant endophytic fungi
PDA	Potato Dextrose Agar
MEA	Malt extract agar
ITS	Internal Transcribed Spacer
PCR	Polymerase Chain Reaction
OTU	Operational Taxonomic Unit
IAA	Indole-3-Acetic Acid
ABA	Abscisic acid
SOD	Superoxide dismutase
CAT	Catalase
MDA	Malondialdehyde
SPAD	Soil Plant Analysis Development Index
MAPK	Mitogen-Activated Protein Kinase
NRPS	Non-Ribosomal Peptide Synthetases
PGPR	Plant Growth-Promoting Rhizobacteria
AMF	Arbuscular Mycorrhizal Fungi
EPS	Exopolysaccharides
GSH	Glutathione
